# Violence against Women with Chronic Maternal Disabilities in Rural Bangladesh

**DOI:** 10.3329/jhpn.v30i2.11312

**Published:** 2012-06

**Authors:** Ruchira T. Naved, Lauren S. Blum, Sadia Chowdhury, Rasheda Khan, Sayeda Bilkis, Marge Koblinsky

**Affiliations:** ^1^icddr,b, GPO Box 128, Dhaka 1000, Bangladesh; ^2^John Snow Inc., Arlington, Virginia, USA

**Keywords:** Emotional violence, Maternal disabilities, Maternal morbidity, Sexual violence, Bangladesh

## Abstract

This study explored violence against women with chronic maternal disabilities in rural Bangladesh. During November 2006–July 2008, in-depth interviews were conducted with 17 rural Bangladeshi women suffering from uterine prolapse, stress incontinence, or fistula. Results of interviews showed that exposure to emotional abuse was almost universal, and most women were sexually abused. The common triggers for violence were the inability of the woman to perform household chores and to satisfy her husband's sexual demands. Misconceptions relating to the causes of these disabilities and the inability of the affected women to fulfill gender role expectations fostered stigma. Emotional and sexual violence increased their vulnerability, highlighting the lack of life options outside marriage and silencing most of them into accepting the violence. Initiatives need to be developed to address misperceptions regarding the causes of such disabilities and, in the long-term, create economic opportunities for reducing the dependence of women on marriage and men and transform the society to overcome rigid gender norms.

## INTRODUCTION

Maternal disabilities with long-lasting consequences have various adverse effects on the health and well-being of millions of women worldwide. Chronic maternal disabilities most frequently occur among women who survive life-threatening, acute maternal complications and are most widespread in resource-poor countries where maternal health services are often inadequate and of low quality (1-3). Within these contexts, acute maternal morbidities have been reported among economically-disadvantaged populations, particularly those living in rural settings with less access to professional obstetric services (1,4,5), although, as reported in this special issue of JHPN, their richer counterparts have more acute maternal morbidities that are clinically diagnosed possibly because they use services more (6,7). In South Asia, acute maternal morbidities are also linked to patriarchal social structures and the low status of women who typically have limited access to skilled healthcare providers (6-8). Despite the concentration of acute maternal morbidities and, hence, also chronic maternal disabilities in resource-poor countries, the social consequences that they may provoke, including violence, have received little attention.

Chronic disabilities resulting from severe acute complications include but are not limited to the following: uterine prolapse—sliding of the uterus from its normal position in the pelvic cavity into the vaginal canal; stress incontinence—an involuntary loss of urine during physical activity; and vesicovaginal fistula (VVF)—an abnormal opening between the bladder and the vagina. These chronic conditions typically trigger a host of co-morbidities, increasing the overall suffering of the affected women (9-12). The co-morbidities include various health and functional problems (13-16). Uterine prolapse, for example, can lead to chronic backache, urinary problems, pain during sexual intercourse, and complications in pregnancies (17). Stress incontinence can cause leakage of urine during intercourse and orgasm (18,19), difficulty in achieving orgasm (10), dyspareunia, vaginal dryness, and decreased libido (11). Victims of VVF suffer from urinary incontinence, which makes them vulnerable to urinary tract infections, vaginitis, excoriation of the vulva, narrowing of the vagina, secondary amenorrhoea, and the inability to carry a foetus to term even after repair (10).

The combination of the disabilities and their co-morbidities can also impact on the ability of women to carry out household chores and partake in wage labour, to maintain an active social life, and to engage in sexual intercourse (20,21), dramatically altering the quality of life of the affected women. The psychosocial and physical changes in women suffering from such disabilities and co-morbidities have been shown to affect relationships with their partners, communities, and the society at large (9,12,14,22,23). A study examining the psychosocial consequences of Bangladeshi women living with fistula found that 33% had reported difficulty in maintaining a sexual relationship, with 50% reporting a significant decrease in libido, 59% a reduction in the frequency of coitus, and 45% a delay in experiencing orgasm (24). Moreover, 52% of husbands expressed a loss in sexual pleasure with their wives.

Social consequences of women with stress incontinence and fistula commonly mentioned in the literature include social isolation, restrictions on religious practices, marital separation and dissolution (25), rejection by family and friends (4,22), poverty (4), begging, prostitution (1,4), and suicide (12). Although women living in impoverished settings suffering from chronic maternal disabilities presumably have a lot in common, their life experiences are context-specific, reflecting their roles in the family and the community and guided by specific ideologies, cultures, and social structures that influence their acceptance in society.

### Chronic maternal disability and violence in Matlab, Bangladesh

Located in South Asia, Bangladesh is a patriarchal society where women have limited education and participation in the labour force. Most women rely on their marital union for economic support in absence of the means to live independently outside the marriage. These factors contribute to their persistent low status in Bangladesh. Succession in Bangladesh is organized along the patrilineal lines; consequently, biological paternity of the child becomes a crucial social issue that necessitates surveillance and control over women's sexuality and reproduction (26,27). Gender roles are clearly delineated (26), with men and women functioning in separate spheres. Typically, men spend more time outside the household while women are relegated to the domestic sphere where their roles involve carrying out household chores, caring for family members, providing husbands with unlimited sexual access (28), and conceiving and bearing children (29).

Within such a context in Bangladesh, it is not surprising that attitudes favouring violence against women are common, and the reported prevalence of such violence is high (28,30,31). According to the Bangladesh Demographic and Health Survey (BDHS) 2007, 49% of ever-married women of reproductive age are physically abused by their husbands, with 18% being victims of sexual violence inflicted by their husbands (32). The Bangladesh component of a multicountry study on women's health and violence against women conducted in 2001 in the same rural area found rates of within-marriage sexual violence as high as 50% (28). Results of this same population-based study showed that 79% of women in this rural site condoned one or more reason(s) for wife-beating (28). A majority of women indicated that women must respond to their husbands' sexual desire, and 29% believed that violence is justified if a wife refuses her husband's sexual desire (28).

Given this tendency towards violence relating to a couple's sexual life and its high acceptance, such violence would potentially occur also if a chronic maternal disability interfered further with sexuality.

The present study carried out in a rural area of Bangladesh attempted to address some gaps in the literature, providing in-depth descriptions of violence perpetrated by husband, family, and community members against women suffering from chronic disabilities, such as uterine prolapse, stress incontinence, and fistula. Qualitative data were collected from women to understand how these conditions affecting female sexual organs and their ability to fulfill gender role expectations might lead to violence in a society where high rates of violence against women are already reported.

## MATERIALS AND METHODS

### Study site

The study was carried out during November 2006–July 2008 in Matlab, Bangladesh. Matlab, situated in a rural setting, 50 km southeast of the capital Dhaka, is the home of a longstanding health and demographic surveillance system maintained by the International Centre for Diarrhoeal Disease Research, Bangladesh (icddr,b). The inhabitants of Matlab are generally poor, with agriculture and day-labour being the primary sources of income. Participation of women of reproductive age in the labour force is only 11%. While literacy and educational levels are rapidly improving as rates of child education increase, 27% of women in Matlab aged over seven years have had no formal education. Only 12% of women aged 15-49 years had education beyond tenth grade whereas 20% of males in the same age-range had the same level of education (33). The majority of residents are Muslim, with a minority Hindu population. Women's lives are centred around the household sphere, and movement far from home is often restricted. This impacts on exposure of women to information and limits their understanding of and access to healthcare.

### Methods and study subjects

The research was part of a broader study designed to examine the social consequences of chronic maternal disabilities women experience. Specifically, we aimed to identify women who had one of the three types of chronic maternal disability—fistula, stress incontinence, or uterine prolapse for at least five years—with the initial goal of interviewing 10 women experiencing each one of these conditions. To identify the women with these conditions, a screening tool was developed and administered by the community health workers during their routine visits to households. Using the tool, 64 women were identified who potentially met the selection criteria. Further in-depth discussions allowed us to reduce the number of probable cases, with some women excluded because they did not meet the study criteria for the minimum number of years they had suffered from the disability. Eventually, 23 women physically examined by a female physician were confirmed to have one of the disabilities under study. Through this approach, three women with stress incontinence, four women with fistula, and 16 women with uterine prolapse were identified. Among four women with uterine prolapse, one refused to participate in the study, and three were not approached as we had 12 women who could participate instead of the target sample of 10 for uterine prolapse. We decided to include a larger number of women with prolapse to increase the overall sample-size. Of the 12 women suffering from uterine prolapse, three had first-, seven had second-, and two had third-degree prolapse.

We carried out in-depth interviews with these 19 women. Various topics were explored relating to their condition, including understanding of the disability, associated physical consequences (co-disabilities), causal explanations, treatment-seeking behaviour, reactions by family members and the broader community, strategies employed to cope up, self-perceptions and psychosocial well-being, and exposure to violence. Data collected from two women were excluded from further analysis as they were not asked about violence. Violence-related questions focused on the forms of violence inflicted due to chronic maternal disability, specific acts of violence, and the perpetrators of these violent acts. If the woman reported being exposed to any form of violence due to her condition, she was asked to describe an event in detail; additional questions were asked about when these acts started, their severity and frequency, and how the acts of violence affected her relationship with the perpetrator(s) and other family members.

### Data-collection procedures

Five female researchers with training in anthropology and extensive experience in qualitative data-collection carried out the interviews in the local language—Bangla. Interviews were administered, either inside the household or in the family-yard, and questioning was terminated and scheduled for a later time if privacy could not be maintained. Initial interviews lasted for 2 to 2.5 hours, and each respondent agreed to attend one or two follow-up visit after the initial interview. Subsequent interviews were designed to fill gaps in the data and explore information in greater depth. Interviews were tape-recorded and subsequently transcribed.

### Analysis of data

Transcribed data were entered and coded according to the main study themes in ATLAS.ti, a text-organizing software. Content analysis was used for identifying and comparing the trends of key concepts in the coded data according to the condition the respondent had.

### Ethical procedures

Verbal informed consent was obtained from all the respondents before participation in the study. The Ethical Review Committee of icddr,b approved the study. Medical treatment was offered to all women included in the study. The names of interviewees were replaced by pseudonyms in the data files and in this paper.

## RESULTS

### Background characteristics

The background characteristics of the women interviewed are presented in Table 1. At the time of interview, six of the 17 women were beyond reproductive age, and only three were aged less than 40 years. Most (n=16) had lived with the condition for 11 years or more. Most (n=14) women in this sample had no formal education. Two women had been abandoned by their husbands, and the majority (n=16) had more than one child. According to wealth ranking based on factor analysis of household assets conducted by icddr,b on all households in Matlab, nine women came from relatively high, two from middle and six from low socioeconomic strata. The study participants were predominantly Muslim (n=15).

**Table 1.** Background characteristics of women interviewed (n=17)

Characteristics	Number
Age (years) of women	
<40	3
40-44	4
45-49	4
50+	6
Education of women	
None	14
Primary (Grade 1-5)	2
Secondary (Grade 6-10)	1	
Marital status	
Currently married	13
Widowed	2
Abandoned	2
Living children	
0	2
1	1
2+	14
Religion	
Muslim	15
Hindu	2
Socioeconomic status	
Low	6
Medium	2
High	9
Duration (years) of condition	
1-10	2
11-20	10
21-30	4
Unknown	1

**Table 2.** Emotional violence due to chronic maternal morbidity

Chronic maternal morbidity	Emotional violence*				Women who reported emotional violence (n=13)	Women with chronic maternal morbidity (n=17)
	*Khota/ tishara* (teasing, castigating, ridiculing, jeering) (n=11)	Verbal abuse (n=5)	Threat of another marriage/abandonment/divorce(n=3)	Blaming (n=2)		
First-degree prolapse	1	1	-	-	1	2
Second-degree prolapse	5	2	1	2	5	6
Third-degree prolapse	1	1	-	-	1	2
Stress incontinence	2	1	-	-	3	3
Fistula	2	-	2	-	3	4

*Multiple responses

### Violence due to chronic maternal disabilities

The results in this section are presented by forms of violence—emotional, sexual, and physical. Within each form of violence, different acts of violence are presented, by trigger and the perpetrator.

#### Emotional violence relating to chronic maternal disability

Emotional violence in response to the disabilities was high. Seven of the 10 women with prolapse were subjected to emotional violence (Table 2). Two women who did not experience emotional violence concealed their condition from others. The other woman with no emotional violence told people that her condition was due to engaging in heavy work immediately after childbirth, and she convinced her husband that the prolapse was caused by having intercourse too soon after the delivery and, therefore, he was responsible. All the three women with stress incontinence experienced emotional abuse, as did three of the four women suffering from fistula.

The main types of emotional abuse were: teasing (*khota, tishara*), demonstration of anger, verbal abuse (*gali-galaj*), threat of abandonment or divorce, and remarriage. The inability to work or to carry out work properly, the inability to provide sexual satisfaction, and verbal conflict were the main triggers for such abuse. Perpetrators included in-laws, husbands, and neighbours. Table 3 shows acts of emotional violence by condition and perpetrator.

##### Teasing, castigating, ostracizing, treating with disgust, putting down, and blaming for the condition

Eleven of the 17 women experienced *khota* or *tishara* (teasing). When recounting their exposure to emotional violence, the word *khota* was mentioned 40 times by the sample women while the word *tishara* was mentioned six times. In-laws in six cases and neighbours in seven cases most commonly teased, castigated, and treated women with a chronic maternal disability with disgust. In three cases, husbands also teased or castigated their wives. Hasina, a woman suffering from second-degree prolapse for the last 13 years, said:

People (in-laws and neighbours) teased me saying, “Look, she has an egg (*dim/anda*) hanging out.” The elderly women cautioned them against teasing me saying, “… If you tease her, you'll also have it (the uterus) out” (Hasina with second-degree prolapse; age 43 years; low socioeconomic status).

Nilu, who suffered from stress incontinence for 20 years, reported being ridiculed, treated with disgust, and called ‘*mutuni-chhutuni’* (a person who leaks) by the neighbours. She said:

The people in the community who know about my condition laugh at me. They treat me with disgust (*ghinna-minna*). I avoid them (Nilu with stress incontinence; age 42 years; high socioeconomic status).

Similarly, a fistula patient named Amena was ridiculed by her in-laws, neighbours, and even the children in the village. They called her names, such as *‘peshap koroni’*, ‘*mutuni’*, i.e. a person who leaks, or ‘*nekrapinda thahsolo’*, ‘*chhalapinda thahsolo’,* a person who uses cloth to prevent urine from dripping.

Saleha, who suffered from second-degree prolapse over the past 10 years, had to put up with *khota* ever since she developed the condition. The following narrative by her illustrates why prolapse is perceived as shameful and how people tended to blame the woman for the condition. Saleha explained:

My daughter-in-law would say, “… You have it hanging out because you are bad. This would not have happened to you if you were a good person.” She freely expresses her disgust (*ghin*), and I feel ashamed (*shorom*).

Saleha continued:

… my sister-in-law said, “You had *honadana* (sex) with your husband while your uterus was still raw (*kacha narh*) and, thus, your *hoshka* (uterus) got out.” … She used to say such things, and everybody in the house heard her (Saleha with second-degree prolapse; age 53 years; low socioeconomic status).

Women were also blamed for social consequences associated with the condition, such as marital dissolution.

##### Emotional abuse relating to household chores

Five women suffering from a disability reported being exposed to *muck larha, gali; gali-galaj* (verbal abuse, mentioned by four respondents) and demonstration of *rag, raga-ragi* (anger, mentioned by four respondents) by family members due to their inability to carry out household chores fully and/or properly. Despite common knowledge that heavy work may aggravate their condition and cause hardship, eight of the 17 women were forced to continue to engage in chores demanding physical strength to keep the in-laws and their husbands happy and, thus, save their marriage. In one case, if any household member provided support, the father-in-law would intervene and insist that the woman with the condition should not receive assistance (see Case Study).

Rupa's case was also very telling. She had her first child at the age of 12 years and developed second-degree prolapse after delivery. The marital family aimed to get rid of her, despite her attempts to carry out all the household chores. Rupa vividly described emotional abuse perpetrated by her husband and mother-in-law for allegedly not being able to carry out household chores. She said:

I cooked and carried out all the other household chores starting from the seventh day after delivery. … I was determined to save my marriage by working hard. I did a lot to make my mother-in-law and husband satisfied. I cooked, washed, collected fuel, and did all the other work. … I used to be afraid that when I lifted the big rice-pot or the pitcher, my uterus would come out altogether. It used to feel so heavy but I still continued working. I did not drop any work. I did not ever refuse my mother-in-law or my sister-in-law any work. … Still they told me, “You won't do. You don't know how to work.” … Actually, they had already made up their mind not to keep me anymore. When I asked my husband why he wouldn't keep me, he said, “You cannot work—you cannot work properly.” … My mother-in-law said, “You need to earn your food by working.” I told her I'd stay even if I need to earn my food by working.” … When I got divorced, they (the in-laws) told the *shalish* (the informal village court) that they wanted to get rid of me as I could not work.

**Table 3.** Acts of emotional violence due to different chronic maternal morbidities by perpetrator

*Khota/tishara*										
Category	Husband (n=3)	Sister-in-law (n=6)	Daughter-in-law (n=1)	Co-wife (n=1)	Neighbours (n=7)	Motder-in-law (n=0)	Fatder-in-law (n=0)	Motder (n=1)	Women who re-ported emotional violence (n=11*)	Women with chronic mor-bidity (n=17)
First-degree prolapse	1	-	-	-	-	-	-	-	1	2
Second-degree prolapse	-	4	1	1	3	-	-	1	5	6
Third-degree prolapse	1	-	-	-	1	-	-	-	1	2
Stress incontinence	1	1	-	-	1	-	-	-	2	3
Fistula	-	1	-	-	2	-	-	-	2	4
Verbal abuse										
Category	Husband (n=5)	Sister-in-law (n=1)	Daugh-ter-in-law (n=0)	Co-wife (n=0)	Neighbours (n=1)	Mother-in-law (n=1)	Father-in-law (n=1)	Mother (n=1)	Women who re-ported emotional violence (n=5*)	Women with chronic mor-bidity (n=17)
First-degree prolapse	1	-	-	-	-	-	1	-	1	2
Second-degree prolapse	2	1	-	-	1	1	-	1	2	6
Third-degree prolapse	1	-	-	-	-	-	-	-	1	2
Stress incontinence	1	-	-	-	-	-	-	-	1	3
Fistula	-	-	-	-	-	-	-	-	-	4
Threat of another marriage/abandonment/divorce
Category	Husband (n=3)	Sister-in-law (n=2)	Brother-in-law (n=1)	Co-wife (n=0)	Neighbours (n=0)	Mother-in-law (n=1)	Father-in-law (n=0)	Mother (n=0)	Women who re-ported emotional violence (n=3*)	Women with chronic mor-bidity (n=17)
First-degree prolapse	-	-	-	-	-	-	-	-	-	2
Second-degree prolapse	1	1	1	-	-	1	-	-	1	6
Third-degree prolapse	-	-	-	-	-	-	-	-	-	2
Stress incontinence	-	-	-	-	-	-	-	-	-	3
Fistula	2	1	-	-	-	-	-	-	2	4
Blaming										
Category	Husband (n=0)	Sister-in-law (n=1)	Daugh-ter-in-law (n=1)	Co-wife (n=0)	Neighbours (n=1)	Mother-in-law (n=0)	Father-in-law (n=0)	Mother (n=0)	Women who re-ported emotional violence (n=2*)	Women with chronic mor-bidity (n=17)
First-degree prolapse	-	-	-	-	-	-	-	-	-	2
Second-degree prolapse	-	1	1	-	1	-	-	-	2	6
Third-degree prolapse	-	-	-	-	-	-	-	-	-	2
Stress incontinence	-	-	-	-	-	-	-	-	-	3
Fistula	-	-	-	-	-	-	-	-	-	4

*Multiple responses

The result of the *shalish* did not go in favour of Rupa, and she got divorced nine months after she developed prolapse. Rupa was separated from her child and ended up living with her natal family. Over time, her condition deteriorated, and her ability to work reduced. Today, she is emotionally abused even by her mother for not fulfilling expectations in relation to the amount of work she is supposed to accomplish. She gave the following example:

Yesterday, I collected some rice seedlings, and then I had to quit due to backpain. … My mother scolded me (*mukh larhsey*), “So, you find it difficult to work but not to eat” (Rupa with second-degree prolapse; age 47 years; high socioeconomic status).

##### Emotional abuse relating to inability to satisfy husbands' sexual needs

Leakage of urine or discharge during or after intercourse (4 cases), painful intercourse (3 cases), problems in penetration (4 cases), lack of interest in sex (3 cases), and white discharge (14 cases) were commonly-reported problems encountered by the women. Five husbands verbally abused their wives and threatened them for divorce or abandonment due to adverse consequences on their sexual life. In Rupa's case, the inability to satisfy her husbands led to marital dissolution on two occasions. Sexual problems associated with prolapse and the subsequent emotional violence that ensued were described by Rupa as follows:

He (her husband) used to say, “because of this (uterine prolapse), I don't enjoy sex as I did earlier. What's the use of keeping you?” … Due to my prolapse (*poddorog*), he could not penetrate properly. I did not like it as well. I used to have pain. I wouldn't have pain when he first penetrated but the pain would come shortly afterwards. I used to tell him, “it hurts”. Then he would move away. … My husband told me, “if I cannot sleep with you what is the use of keeping you? I'll divorce you” (Rupa with second-degree prolapse; age 47 years; high socioeconomic status).

##### Emotional abuse relating to treatment expense

While most families could not afford to pay for treatment of the condition and associated co-morbidities, three did have the resources but did not provide the women with necessary medical treatment. It was not quite clear whether this was due to lack of information or an unwillingness to bear the expenses. This situation is illustrated in Case Study of Forida (Box).

**Box. Forida's story**

Forida came from a financially well-off rural family. She got married at the age of 18 years. Her husband, who was a farmer at the time of the marriage, started working abroad later and vastly improved the financial condition of the family. Forida started suffering from uterine prolapse 21 years ago after her first delivery. Over time, she also developed stress incontinence. She had three children, and with each delivery, her condition deteriorated. Although she immediately informed the family members about the uterine prolapse, she tried to conceal the problem of incontinence fearing backlash (e.g. deterioration of her relationship with her husband and another marriage by husband) of reporting so many health problems.

Forida's father-in-law used to verbally abuse her for treatment expenses and for her inability to carry out household chores. For a long time, Fordia's husband, however, was very supportive and tried his best to get her cured. For the treatment, the family had to sell livestock and agricultural produce kept for sale during crisis. Yet, Forida's husband never asked her to bring money from her natal family for treatment. However, later the need to support long-term treatment and tolerate the inconveniences of the disabilities made him impatient, fuelling his anger, making him refuse to give money for treatment, making derogatory comments about her condition, and causing him to become violent towards his wife.

Forida's condition had an adverse impact on her sexual life, including exposure to sexual violence. She experienced lot of pain during intercourse. Her husband used to have difficulty inserting his penis into the vagina due to the prolapsed uterus. She used to leak during intercourse getting her husband wet. Her husband used to call her names, curse her, and slap her when he experienced such discomfort during sex. He used to force Forida for having anal and oral sex or sex between her breasts, with a threat of divorce. Before she developed the conditions, she could refuse to have such acts of sex. To save her marriage, she submitted to such sexual demands even though she felt with disgust.

#### Physical and sexual violence relating to chronic maternal disability

Eight of the 17 women reported experiencing sexual violence, which they associated with their conditions (Table 4). Three of these women could not refuse having sexual relations with their husbands, apprehending repercussions, including a negative outcome of their relationship. Thus, they allowed their husbands sexual access even when they were not interested. Six women were physically forced and/or coerced to have sex even when they expressed their unwillingness. One woman was coerced into engaging in sexual activities she considered unsavory, such as anal and oral sex. In two cases, unwillingness to comply with husband's sexual demands eventually led to marital problems.

Disclosure of pain during intercourse was one of the reasons for the breakdown of Rupa's first marriage and deterioration of the relationship with her second husband. Rupa's situation portrays the marital problems that women apprehend, forcing them to conceal their health problems, or to delay disclosure of the problems to their spouse. An extreme example of silence is Saleha who had a prolapse after the birth of her sixth child. She gathered the courage to inform her husband about her uterine prolapse 20 years after she had developed the condition and had given birth to three more children. Saleha told the interviewer:

I used to get hurt when we had sex. … But I didn't tell him anything fearing that he would have negative notions about me. … I was afraid he'd tell me that he wouldn't keep me or that he'd marry again. This is why I did not want to tell him about my condition or about the pain (Saleha with second-degree prolapse; age 53 years; low socioeconomic status).

Apart from Rupa and Saleha, five other women talked about their condition and the discomfort it caused in having sex with their husbands. However, this did not preclude them from engaging in sex. Suffering with stress incontinence, Nilu talked about trying to resist sex but eventually submitted to the wishes of her husband. She said:

About 4-5 years ago, I realized that my pain increases when I have sex. … But will men listen if we refuse them sex? He used to say, “You have to let me have sex.” So, I used to surrender (Nilu with stress incontinence; age 42 years; high socioeconomic status).

Shahana had pain during intercourse and lacked interest in sex which she attributed to her second-degree prolapse. Any attempt to resist having sex with her husband was met with physical aggression, involving slapping and forcing her to comply. This continued for about three years after she developed the condition; subsequently, she stopped protesting.

In Forida's case, sex was physically forced at times (see Case Study). She was also forced to partake in undesired sexual acts, which she could not resist out of fear of divorce.

### Consequences of violence relating to chronic maternal morbidity

#### Silence about chronic maternal disability induced by apprehension of stigmatization and violence

When the women were asked about the consequences they anticipated at the time the disability developed, the most common responses were that the condition would cause *shorom* or *lojja* (stigma), elicit *khota* or *tishara* (teasing, castigating, ridiculing, jeering), and jeopardize the marital relationship due to their inability to satisfy their husband's sexual needs and carry out household responsibilities. The number of times the terms *shorom*, *lojja*, *khota,* and *tishara* appeared in the data indicates their importance for the women. Stigmatization was a recurring theme in the women's narratives of what they apprehended. Thus, *shorom* was mentioned by seven women 19 times while *Lojja*—another word for stigmatization—was mentioned by three women five times. *Khota* and *tishara* were the next most frequently mentioned consequences that women were apprehensive of when they discovered their conditions. Seven women mentioned apprehending *khota,* using the term 15 times, and two women mentioned the term *tishara,* referring to it five times. Two women mentioned fear of jeopardizing the marital relationship.

**Table 4.** Sexual violence due to chronic maternal morbidity

Chronic maternal morbidity	Sexual violence*			Women who reported sexual violence (n=8)	Women with chronic maternal morbidity (n=17)
	Acceptance of sexual advances out of fear (n=3)	Forced sex (n=7)	Imposition of unacceptable sexual acts (n=2)		
First-degree prolapse	1	1	1	1	2
Second-degree prolapse	2	2	-	3	6
Third-degree prolapse	-	1	-	1	2
Stress incontinence	-	2	-	2	3
Fistula	-	1	1	1	4

*Multiple responses

Our findings showed that *khota* (teasing) was used as a weapon to denigrate women with maternal disabilities, especially during verbal conflict. Women were particularly afraid of being ridiculed by the neighbours and in-laws for the condition, which caused *shorom* or shame. For example, Rakhi, a woman with stress incontinence said:

Our neighbours do not know anything about this. I did not tell them. … If people can find a deficiency, they will castigate me (*khota dibo*). During quarrels, they will take the upper hand and stigmatize me (*shorom dibo*). This is why I don't discuss this with anyone (Rakhi with stress incontinence; age 28 years; high socioeconomic status).

*Khota* was considered particularly effective in belittling the woman because it relied on the social stigma associated with the chronic condition and accompanying co-morbidities, which according to cultural norms, dishonoured the woman and undermined her value. Thus, for example, a woman having incontinence was considered imperfect and impure due to her inability to control leaking urine. A woman with uterine prolapse was perceived to have failed to adhere to social norms involving abstaining from sex for 40 days after childbirth and, thus, engaging in inappropriate behaviour only followed by a ‘bad’ person. Similarly, fistula was perceived to be caused by intercourse during pregnancy, which is frowned upon.

With onset of the condition, the women were apprehensive of their future sexual lives. Rupa, who had second-degree prolapse as mentioned earlier, said:

I thought *poddonarh* (uterine prolapse) would give out a bad smell that people wouldn't be able to tolerate. Also, I was not sure whether I could have intercourse with my husband anymore.

Another woman with second-degree prolapse said:

If he (the husband) could not have sex the first day, then the second day, and then the third day again, he would go elsewhere for sex (Shahana with second-degree prolapse; age 47 years; low socioeconomic status).

Saleha, another woman with second-degree prolapse, said:

I did not tell him when I developed this (uterine prolapse). … I was scared that (if he came to know) he might tell me that he wouldn't keep me and would marry again. This is why I did not want to tell him (Saleha with second-degree prolapse; age 53 years; low socioeconomic status).

Forida, who had first-degree prolapse, offered a different perspective, sharing fear that merely having the condition may encourage her husband to marry again.

#### Other consequences of violence due to chronic maternal disabilities

Most women mentioned feeling bad, hurt (*kharap, koshto*), and ashamed (*lojjito*) when they were subject to abuse associated with their condition. Symptoms typical of mental health problems were apparent in Forida's narrative of the consequences of the abuse she experienced. She said:

I felt helpless and bad. I feel restless whenever I remember what I was told or how I was treated. I feel dizzy. I lose my peace of mind. I can sleep very little and cannot think clearly. I feel suffocated. I feel better when I am among other people (Forida with first-degree prolapse; age 41 years; high socioeconomic status).

## DISCUSSION

In our study, most of the women living with a chronic maternal disability—stress incontinence, uterine prolapse, or fistula—were subjected to emotional violence, and almost half of them were sexually abused. Their accounts provide insights not only into the specific acts of emotional and sexual violence and their triggers but also their vulnerabilities which make them endure the violence.

The common triggers for violence were the inability of the woman to carry out household chores or satisfy her husband's sexual needs as expected. In addition, any of the three disabilities worked as a trigger for the emotional violence with teasing, castigating, humiliating, putting down, and blaming the woman, highlighting the way in which a physical change, particularly one affecting a woman's sexual organs, manifests stigma, and alters the social status of the woman.

The existing misconceptions regarding the precursors of uterine prolapse and fistula were often based on the premise that the women had engaged in inappropriate sexual behaviour, thus creating an opportunity to blame the affected women for the condition.

Stigmatization and blame towards these women have some common features with the experience of childless women in rural Bangladesh. As described by Nahar, the patriarchal context of the society favours men and readily finds fault in females when things go wrong (34). Both conditions trigger emotional abuse not only by husbands and/or other household members but also by the community. Thus, violence against women with chronic maternal disabilities crossed the boundaries of domestic violence, which is common in Bangladesh and imposed an added layer of abuse by the community.

With sexual violence involving forced sex or acceptance of husband's sexual advances even when women experienced pain or lacked desire to have intercourse, apprehension of physical repercussions and marital dissolution silenced women's lack of sexual desire and/or painful intercourse and forced them to engage in intercourse and put up with undesired sexual acts. In this social context, both men and women believe that a husband has the right to have unlimited sexual access to his wife's body; refusal of sex to a husband is highly unacceptable, justifying physical violence (28,32).

Taken together, the chronic maternal disabilities evoked extreme shame and mental anxiety about the stability of their marriages and the willingness of the extended families (in-laws) to retain afflicted women in their households. Thus, silence about the disability was a primary means to manage living with the disability. The perceived vulnerability of the affected women discouraged them from sharing the condition and the physical discomfort it caused. The social consequences of women living with fistula have also been reported as similar—stigmatization that forces them into silence (35). Although the silence provided women respite from stigmatization, it did not necessarily allow them to avoid violence as they had to put up with demands for the fulfilment of gender role expectations in terms of carrying out household chores, satisfying sexual needs of the husband, and even giving birth to more children causing harm to their health and well-being.

In the Bangladesh context, where the level of violence against women is already high (36,37), rates of female education are low, and women are typically economically dependent on their husbands, the added burden of having a chronic disability jeopardizes a woman's position in the household and broader social structure and increases the likelihood of violence. The added burden of suffering from such a disability and the diminished self-worth that ensues appears to foster more profound sentiments of female inferiority, disempowerment, and a willingness to accept gender-based violence, which needs to be addressed.

These findings underscore the need to develop educational efforts to inform the public about the nature of these disabilities, including the cause and appropriate physical and psychological care for women with such problems. Another option is to develop educational and training programmes that will equip women with practical skills to generate income and become less dependent on husband and family members. Interventions must also be developed to promote sexual rights of women and criminalize sexual violence within marriage. Over the long-term, such efforts may impact on altering the prevailing sexist male attitudes and broader social norms, condoning male dominance.

### Limitations

The sample-size of the study was small and included a disproportionate number of women from relatively-higher socioeconomic strata, who may be less likely to be victims of domestic violence. The study may have missed women even more exposed to violence as they are likely to get divorced and perhaps have left the study area.

As Ellsberg *et al*. have demonstrated, research entirely devoted to violence yields more reporting than if violence is just one of the study components explored (38). In this case, the present study focused more broadly on the social consequences of chronic maternal disabilities. We suspect that recall bias, the silence used for mediating these disabilities, and the reluctance on the part of women to recall negative experiences of the past, and that violence was only one of the focus areas of the study may have contributed to the under-reporting of the abuse inflicted on women.

### Conclusions

This study examining violence inflicted on women due to chronic maternal disabilities highlights yet another layer of injustice and hardship endured by women living in a South Asian context where females play a subordinate role. The results emphasize the need to develop programmatic approaches to: prevent chronic maternal disabilities; educate the family and the community about causes and prevention of these conditions; and enhance opportunities for women outside marriage through education and employment leading to enhancement of their status and rights in the household, community, and broader society, making them less vulnerable to violence and more knowledgeable of their rights as human beings.

## ACKNOWLEDGEMENTS

The study was funded by United States Agency for International Development (USAID) (Grant No. GHS-A00-0300019-00). The authors acknowledge with gratitude the commitment of USAID to the icddr,b's research efforts. They also express their gratitude to the women who provided them with a wealth of information, a fraction of which was used in this study.
